# Enhancing maternal and infant wellbeing: study protocol for a feasibility trial of the Baby Triple P Positive Parenting programme for mothers with severe mental health difficulties (the IMAGINE study)

**DOI:** 10.1186/s13063-018-2869-z

**Published:** 2018-09-10

**Authors:** Anja Wittkowski, Kim Cartwright, Richard Emsley, Penny Bee, Rachel Calam, Catherine Cross, Kathryn M. Abel, Holly Reid

**Affiliations:** 1 Division of Psychology and Mental Health, School of Health Sciences, Faculty of Biology, Medicine and Health, The University of Manchester, Manchester Academic Health Science Centre, Zochonis Building, Brunswick Street, Manchester, M13 9PL UK; 2 Department of Clinical Psychology, Greater Manchester Mental Health NHS Foundation Trust, Laureate House, Wythenshawe Hospital, Southmoor Road, Manchester, M23 9LK UK; 3 Department of Biostatistics and Health Informatics, King’s College London, Institute of Psychiatry, Psychology & Neuroscience, De Crespigny Park, London, SE5 8AF UK; 4 Division of Nursing, Midwifery & Social Work, The University of Manchester, Jean MacFarlane Building, Oxford Road, Manchester, M13 9PL UK

**Keywords:** Randomised controlled trial, Inpatients, Acute psychiatric wards, Mother and baby unit, Perinatal, Parenting, Women, Infants, Admission

## Abstract

**Background:**

There is a strong evidence base for the benefits of parenting interventions for parents without severe mental illness (SMI). As the impact of maternal SMI can be significant on child development, mothers need support to maximise outcomes for themselves and their children. Some mothers with SMI require admission jointly with their baby to a Mother and Baby Unit (MBU), a psychiatric inpatient ward, for assessment and treatment. However, MBUs do not yet offer formally evaluated, evidence-based parenting interventions as a matter of routine. This paper describes a study to investigate the feasibility and acceptability of conducting a randomised controlled trial (RCT) to evaluate a parenting and psychological intervention targeting the mother’s and infant’s wellbeing for mothers admitted to a MBU.

**Methods/Design:**

This study is a multisite, single-blind feasibility trial with half the participants randomised to the Baby Triple P Positive Parenting Programme plus treatment as usual (TAU) and the other half randomised to TAU alone. Self-report and observer-rated assessments are collected at baseline, 10 weeks post-baseline and 6 months post-baseline. Participants are mothers admitted to a MBU in the Northwest of England or the Midlands. Participants are included if they are fluent in English to provide informed, written consent. Our objective is to determine whether we can recruit 66 women, randomise 60, and retain them in the intervention and study, and whether the intervention and study procedures are acceptable. As part of a nested process evaluation, qualitative interview data from trial participants and MBU staff will inform feasibility and acceptability. The feasibility of collecting data required to conduct an economic evaluation of the intervention will also be explored.

**Discussion:**

Although research has been conducted in relation to mothers with severe mental illness and MBUs, to our knowledge, this is the first controlled trial to test the feasibility, acceptability, uptake and retention alongside the potential efficacy of a parenting intervention for this population. This study is essential to examine the contextual challenges involved in this setting with this population and to identify any refinements required.

**Trial registration:**

ISRCTN12765736. Date of first registration: 2 February 2017.

**Electronic supplementary material:**

The online version of this article (10.1186/s13063-018-2869-z) contains supplementary material, which is available to authorized users.

## Background

Given the significant impact of maternal mental illness on child development [1–4], the importance of interventions to promote optimal outcomes for children in the early years has been widely recognised [5–11]. However, in order to raise infants in loving and supportive environments, parents need to feel confident in their parenting skills [12]. Parents, and especially mothers, with good parenting skills feel that they have a good parent–infant relationship. This relationship has been found to influence the infant’s wellbeing, social and academic competence, and emotion regulation abilities [1–4, 13, 14].

Parenting an infant can be a challenge for parents enjoying good mental health, but it can be an even greater challenge for parents who are mentally unwell. Maternal mental illness has profound effects on the woman, her family and children [1–4, 13, 14]. Estimates suggest that 10–20% of women develop mental health problems during pregnancy or within the first year of having a baby [11]. Furthermore, it is estimated that 1% of women have severe mental illness (SMI) or significant mental health difficulties requiring specialist psychiatric services [15, 16]; in 2013, at least 6985 of the 698,512 women giving birth required admission to a Mother and Baby Unit (MBU). Research has shown that perinatal depression, anxiety and psychosis carry an estimated total cost to society of £8.1 billion for each 1-year cohort of births in the UK [11], a cost of approximately £10,000 for each birth. Perinatal psychosis costs approximately £53,000 per case [11].

The UK has been at the forefront of developing inpatient psychiatric units to admit mothers with existing or perinatal onset SMI, jointly with their babies [17]. These mothers present with various difficulties, including (puerperal) psychosis, bipolar disorder, schizophrenia, severe affective illness (such as postnatal depression) and mother–infant relationship difficulties. The importance of specialist perinatal services in the treatment offered to these mothers and their families has been recognised [6, 8, 15, 16, 18, 19]. Despite improvements in mental illness and mother–infant interactions, as yet there are no structured parenting interventions that guide health professionals to help mothers with mental health difficulties strengthen early parenting [20, 21].

The NICE guidelines and reviews recommend psychiatric and psychological interventions for mothers with mental health problems [5–10, 19, 22], because the quality of the mother–infant relationship in mothers with severe mental health difficulties can be poorer compared to mothers with mood disorders [23, 24]. Although research has shown that treating the mother’s symptoms improves her mental health, this improvement does not necessarily directly relate to her interaction with her infant [25]. Interventions using video feedback appear to be useful in improving the mother–infant interaction in mothers with severe mental health difficulties [26, 27], but video feedback focuses only on interactions and, as yet, this intervention has not been shown to be effective for mothers admitted to a MBU. Therefore, there is a need for further research on parenting interventions with a skills training component for mothers who are looking after babies and who experience SMI or severe mental health difficulties (e.g. severe depression or postpartum psychosis) [20, 28].

Parenting interventions help parents learn how to provide appropriate emotional and physical care, so that children can thrive and reach their full potential [29]. Although parenting programmes improve the emotional and behavioural adjustment of children and parental psychosocial wellbeing [30–32], the importance of addressing parenting issues as well as parental mental health needs has been emphasised [33, 34]. Certainly, mentally ill mothers are not inherently poor parents. Indeed, a review and meta-synthesis of 23 studies has identified the importance women with severe mental health difficulties attach to motherhood [35]. However, mothers have expressed that the demands associated with parenting whilst at the same time coping with significant mental health difficulties are considerable [36].

Parenting competence and confidence help parents to balance parenting demands with their own needs [37]. A Dutch study reported increased parental self-efficacy following participation in a parenting intervention for parents with severe mental health difficulties, but these were not parents of infants (less than 12 months old) [34]. Effective interventions focusing on the parenting needs of mothers with significant mental health difficulties, who parent their babies, are needed [20, 33, 38].

### Positive parenting programme

The Triple P system of interventions has impressive theoretical, scientific and clinical foundations [39–43]. Its aims include (1) enhancing parental knowledge and resourcefulness, (2) promoting nurturing, low-conflict environments for children, and (3) promoting children’s social, emotional and intellectual competencies through positive parenting practices.

The Triple P framework offers accessible, multi-level interventions increasing in intensity for parents with different needs, regardless of sociocultural differences, age and gender [39]. Triple P has recently been expanded to families with children aged under 1 year, which provides an opportunity to test the intervention with mothers with severe mental health difficulties.

The Baby Triple P Positive Parenting Programme, designed to be an intervention at level 4 of the Triple P hierarchy of five levels, has been developed to enhance parental knowledge, skills and confidence. It targets three important areas crucial in enhancing maternal and infant wellbeing, namely (1) positive parenting skills promoting secure attachment, reducing maternal and infant distress, (2) improving partner and social support to increase maternal and overall family wellbeing and (3) increasing maternal coping resources to reduce mental health difficulties. Its primary focus is on enhancing maternal parenting competence and confidence, thereby improving the quality of the mother–infant relationship and subsequent attachment, which in turn improves infant and maternal wellbeing.

### Baby Triple P Positive Parenting studies

In preparation for this feasibility study, the chief investigator has led a number of studies related to Baby Triple P. For example, a small pilot study examined the feasibility and acceptability of Baby Triple P in mothers with postnatal depression [44]. The results were in the predicted direction regarding level of happiness, self-regulation, subjective bonding and depression (at post-treatment only). The 12 women who received Baby Triple P completed all sessions, were retained for all follow-up assessments and rated it as highly acceptable.

A second study explored the views of mothers admitted to the Manchester MBU about the perceived acceptability and feasibility of a parenting intervention like Baby Triple P in the setting of a MBU. Using Q-methodology, which is designed to assess opinions and views, all participants agreed on the perceived benefits of Baby Triple P and the MBU environment as suitable to facilitate it. The intervention was viewed as an acceptable and feasible parenting intervention [45]. However, without staff support, the possible implementation of this intervention on the MBU would be difficult. Consequently, we conducted another study with MBU staff [46], which showed that staff also regarded this intervention to be feasible and acceptable for the MBU setting. MBU staff indicated that the intervention could help reduce the ‘bad parent’ stigma often identified in the population of mothers presenting with severe mental health difficulties [35].

Given these encouraging findings, we aim to test the feasibility and acceptability of conducting a randomised controlled trial (RCT) to evaluate the Baby Triple P programme plus treatment as usual (TAU) in mothers with severe mental health difficulties admitted to one of two UK National Health Service (NHS) MBUs. Qualitative data from MBU staff and participants will be used to (1) assess the acceptability of the intervention and RCT procedures and (2) allow the refinement of the approach and intervention in preparation for a larger clinical trial. The overall aim of this feasibility study is to evaluate the Baby Triple P intervention in mothers with severe mental health difficulties in a MBU setting. The objectives of this feasibility RCT are:

1. To examine the feasibility of the design and procedures of a future RCT.
2. To examine the acceptability (to mothers and MBU staff) of the design and procedures of a future RCT.
3. To examine the key cost indicators associated with the intervention.
4. To involve service users to guide and inform the research.
5. To examine the feasibility of (1) the recruitment methods (through the MBU ward staff and consultant psychiatrist), (2) recruiting sufficient numbers of mothers to be able to estimate an effect size for a future RCT, (3) the randomisation procedures in inpatient ward settings, (4) engagement and retention of mothers in the study (including completion of outcome measures) and (5) piloting data collection procedures (including frequency, duration and format of assessments).
6. To examine mothers’ and MBU staff acceptability of Baby Triple P.
7. To determine the best strategies to avoid or minimise possible contamination (e.g. mothers sharing the Baby Triple P workbook with mothers in the TAU group) or log such incidences.
8. To assess the fidelity of Baby Triple P delivery on the MBU.
9. To determine factors associated with engagement of mothers in and adherence to Baby Triple P.
10. To examine attrition rates and causes and explore solutions to attrition.
11. To determine the suitability of the primary and secondary outcomes and their respective measures for a future RCT alongside appropriate follow-up assessment time points.
12. To explore the key cost elements and the appropriateness of the five level EQ-5D (EQ-ED-5 L) as a measure for a calculation of utility of the intervention in this population.

We aim to (1) establish whether the study procedures work and are acceptable to participants and staff so that a future full-scale trial and economic evaluation can follow and (2) explore whether the intervention is perceived to be beneficial in improving maternal parenting confidence, mood and other psychological symptoms, quality of life and mother–baby bonding.

## Methods/Design

### Preliminary pilot work

We conducted two studies to investigate patient (*n* = 15) and staff (*n* = 16) views and opinions regarding the acceptability of an intervention like Baby Triple P being offered on a MBU [45, 46]. These findings alongside information from a pilot study of Baby Triple P offered to mothers with postnatal depression [44] and patient and public involvement consultations were used to inform the treatment and implementation protocol of this RCT.

### Trial design

**Fig. 1:**
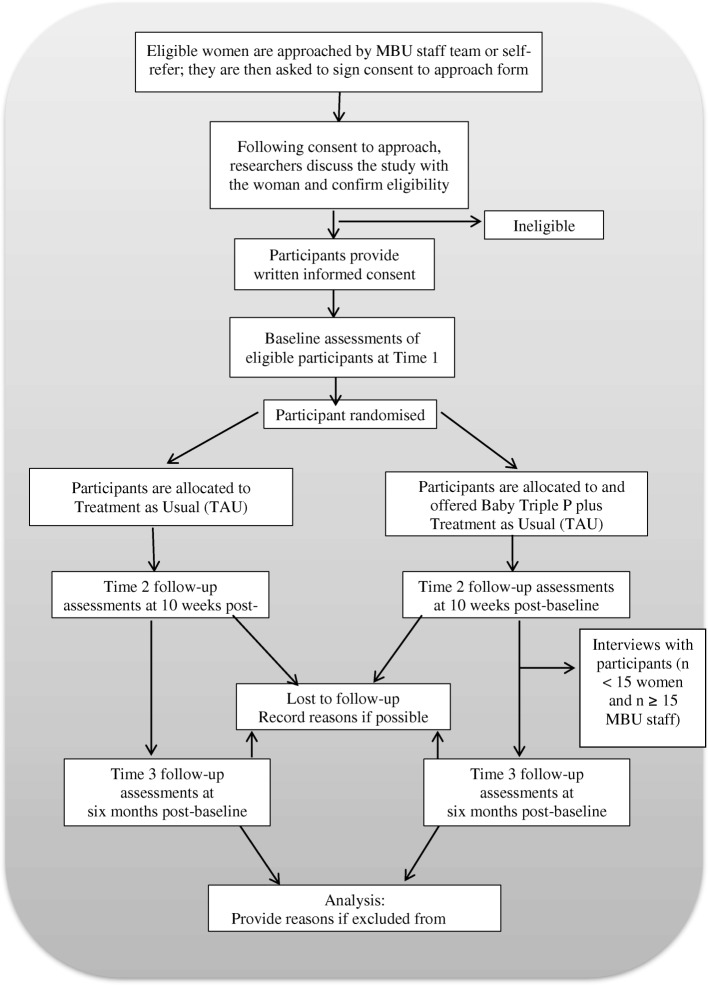
CONSORT diagram showing study design

**Fig. 2:**
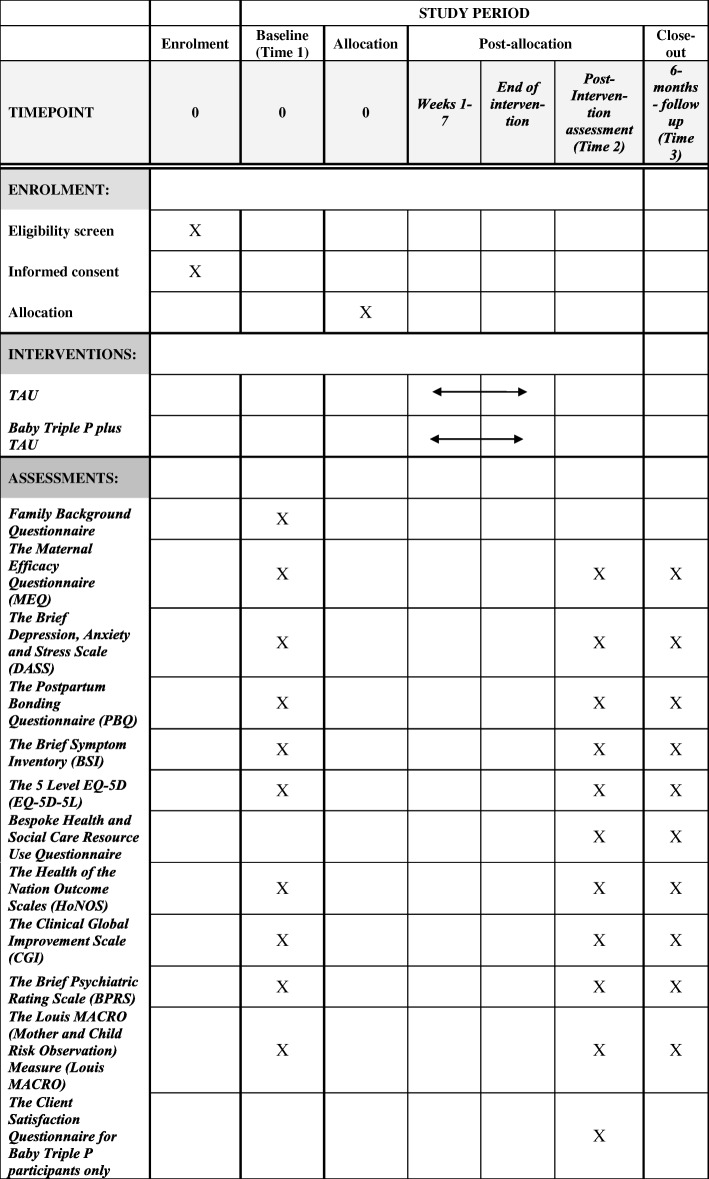
Schedule of enrolment, interventions and assessments

Following the Medical Research Council framework for the development and evaluation of complex interventions [47], this study includes quantitative and qualitative assessment of feasibility and acceptability. It is designed as an individually randomised, multisite, parallel- group, single-blind (outcome assessors) feasibility RCT comparing a parenting intervention (the Baby Triple P Positive Parenting Programme) plus TAU with TAU alone. The trial was designed to be individually randomised because the small number of MBUs would not permit cluster randomisation in a future definitive trial. To mitigate against contamination, participants randomly allocated to the intervention will be asked not to share the intervention workbook and any other information learnt during sessions with other mothers on the MBU. Figure 1 provides an overview of the trial design based on CONSORT guidelines [48], whilst Fig. 2 shows a schedule of study enrolment, interventions and assessments. The recommendations for interventional trials (SPIRIT) checklist [49, 50] is available as Additional file 1.

Assuming a drop-out rate of 10% between recruitment and randomisation in an inpatient setting, a maximum of 66 participants will be recruited and a maximum of 60 will be randomised after completion of baseline measures during their inpatient admission to a MBU. Participants will be randomly allocated, using an independent Clinical Trials Unit (CTU), to TAU or to TAU plus eight sessions of Baby Triple P. Additional sessions can be offered, for example, to conduct an initial assessment and, if required, the intervention can be delivered more flexibly.

Participants are followed up at 10 weeks post-baseline and at 6 months post-baseline. The end of treatment is not fixed but dependent on progress and participants’ capacity to engage, whereas assessment time points are fixed.

### Trial setting

This feasibility study is being conducted in two MBU located in the North West of England and the Midlands, England, UK. MBUs are inpatient psychiatric units for mothers who are admitted jointly with their babies and who require psychiatric nursing care [8, 51]. MBUs in England are commissioned by NHS England. They typically have 4–12 beds and are staffed by specialist perinatal mental health staff, including psychiatrists, nursery nurses, psychiatric nurses and clinical psychologists. Average admission to MBUs in the UK is 7 weeks, ranging from 4 to 12 weeks [21]. The two MBUs are comparable in terms of size and staffing. Both MBUs are located in large cities with socially and ethnically diverse populations.

### Ethics and governance

This trial is supported by the National Institute for Health Research ‘Research for Patient Benefit Programme’ (NIHR RfPB, grant number PB-PG-1014-3505) and sponsored by the University of Manchester (16233). The study has been approved by the NHS National Research Ethics Service via the North West – Greater Manchester South Research Ethics Committee (REC reference number 16/NW/0510). It is conducted following the Good Clinical Practice guidelines in accordance with the principles of the Declaration of Helsinki. The study also has Health Research Authority approval (IRAS project number 188486, protocol number 16233) and Research and Innovation approval by both NHS trusts overseeing the two participating MBUs (e.g. Greater Manchester Mental Health NHS Foundation Trust and Birmingham and Solihull Mental Health NHS Foundation Trust). Trial monitoring is carried out via the Manchester CTU and the trial is also overseen by an independent Trial Steering and Data Monitoring Committee consisting of academics, clinicians, a service user and an independent statistician. Through regular meetings, these committees will be informed of any adverse events and protocol modifications. Procedures are in place to promote data quality (e.g. by ensuring data coded and entered into databases are double checked, etc.). Personal data will be kept confidential and information will be anonymised in the datasets, which will be accessed by the immediate research team only.

Various aspects of the study, such as the wording in participant information sheets and future dissemination plans, will also be informed by our service user group (or Patient and Public Involvement group). This service user group consists of up to six mothers with past experiences of severe mental health problems and/or inpatient admission to a MBU.

### Inclusion and exclusion criteria for participants

Participants are women who are either pregnant or admitted jointly with their infant to a MBU for the assessment and treatment of their severe mental health problems. As the intervention was designed to be universally applicable to parents and caregivers, all eligible participants can take part irrespective of their admission status (voluntary or involuntary, i.e. detained under the Mental Health Act) and their current baby care. Participants are eligible to take part if they are aged 18 or over, have at least one infant aged from 0 to 12 months who they parent or are going to parent, or if they are in the third trimester of pregnancy and are expected to reside on the MBU following delivery. Participants have to be able to comprehend and understand English and provide written informed consent.

Participants are excluded if their discharge has been scheduled within 7 days from the date of recruitment or if their infant will be removed from their care on a non-temporary basis. Participants whose current symptoms seriously compromise their ability to concentrate on the assessments or intervention sessions or who show severe personality disorder traits, including significant self-harming behaviours (such as attempting suicide), will not be considered for inclusion until their symptoms have stabilised as judged by their psychiatric team.

### Nested process evaluation: inclusion and exclusion criteria for participants and staff

As part of a nested process evaluation, we will undertake a qualitative study of staff and patient experiences and views of Baby Triple P and a sample of participants (*n* < 15 women and *n* ≥ 15 MBU staff) will be interviewed. Interviews will explore participants’ and MBU staff experiences and expectations of the study procedures and the intervention alongside its content. Facilitators and barriers to implementation of this type of intervention within a MBU setting will be identified.

Participants (*n* < 15) who were allocated to the intervention arm plus TAU, irrespective of subsequent intervention engagement, will be asked to take part in interviews for the nested qualitative aspect of this study. The same inclusion and exclusion criteria as above will apply.

MBU staff participants (*n* ≥ 15) will also be recruited if they are permanent full-time or part-time members of the MBU staff team and have worked on the MBU for at least 6 months. Psychiatry and medical MBU staff will also be recruited if they have worked on the MBU for at least 4 months. Separate participant information sheets and consent forms for this aspect of the study will be used; similar consent procedures outlined below will apply.

### Recruitment and consent procedures

Recruitment is over an 18-month period from November 2016. Posters are displayed on the MBU to alert potentially eligible patients that this study is being undertaken. MBU staff also identify eligible patients and offer them a Consent to Approach form (a form which gives written consent for the research team to approach patients with information about the study) to sign if they are interested in hearing more about the study. Eligible patients who provide consent (to be approached by the research team) are then approached in person by the researcher who (1) provides them with a Participant Information Sheet outlining the study procedures and requirements, (2) explains the research to them and (3) gives them the opportunity to ask questions. Eligible patients are required to take at least 24 h to think about whether they would like to take part in the feasibility trial. As the MBU is an inpatient psychiatric unit, some participants may require more time to consider their participation, and this will be allowed. After 24 h, or when participants indicate that they are ready to discuss their possible participation further with the researcher, or if the participant is happy to take part, written informed consent is obtained. If they do not wish to take part, they are thanked for their time and asked if they are happy to share their reason for not wishing to take part, but are not obliged to do so. Furthermore, the project manager checks for continued consent with participants when (1) informing participants which arm they have been allocated to, (2) when patients are discharged from the ward, and (3) before and at the beginning of follow-up assessments. A copy of the consent form is given to the participant to keep, one is placed in the clinical notes and another in the investigator site file. Consent forms are retained as records.

After participants have provided written consent to take part in the study, we ask them to complete a Consent to Contact form and to provide their email addresses and telephone numbers so that the research team can contact them (1) to remind them of any study assessment or intervention appointments or interview times, (2) to forward a summary of the findings if they indicate on the consent form that they wish to receive this and (3) to contact them about taking part in other studies within the programme of research if they indicate on the consent form that they agree to this.

Each participant has the right to withdraw from the study at any time. In addition, the research team may decide to discontinue a participant from the study at any time if this is deemed to be necessary for one of the following reasons: ineligibility (either arising during the study or retrospectively), significant protocol deviation, significant non-compliance with the study requirements, withdrawal of consent and loss to follow-up. The reason for withdrawal will be recorded if participants are happy to share this but they are not obliged to do so.

All participants are reimbursed at the end of the study with £30 to thank them for their time and contributions. They are also offered a certificate and a list of useful contacts/organisations for additional support.

### Randomisation

After written consent has been obtained, participants and MBU staff are asked to complete the first battery of outcome measures (i.e. the baseline assessment) before participants are randomly allocated to either TAU or TAU plus Baby Triple P. Randomisation is provided by an independent CTU with an allocation ratio of 1:1 and randomised permuted blocks of size 4 and 6, and is stratified by MBU site. Once the Manchester CTU informs the project manager of the arm to which the participant has been allocated, participants are informed of the randomisation outcome and provided with a randomisation leaflet outlining what they can now expect.

### Blinding

Procedures for maintaining blindness have been developed for this trial to minimise the risk of the research assistant becoming unblinded to treatment allocation. Participants are reminded before and at the beginning of follow-up assessments not to reveal their treatment allocation to the research assistant. The trial project manager, not blinded to treatment, manages all follow-up assessments (including a telephone call to participants before the research assistant contacts participants to arrange follow-up assessments to check continued consent and remind participants not to reveal their treatment allocation to the research assistant) and monitors breaches to blindness. The research assistant is asked to indicate at the beginning of each follow-up assessment which treatment arm they think participants have been allocated to and give a confidence rating and reason for this judgement; this information is recorded in the Case Report Forms and monitored. When breaches occur prior to follow-up appointments, an alternative blinded research assistant will undertake subsequent data collection.

### Intervention and its delivery

The Baby Triple P Positive Parenting Programme consists of eight sessions (estimated to be delivered over 8–10 weeks) by trained facilitators. All participants allocated to receive this intervention will be given the Baby Triple P workbook to keep and share with their partner and family.

The first four sessions are usually delivered face-to-face on an individual basis by drawing on the information provided as presentation slides or in the handbook. These sessions are designed to enhance the knowledge, skills and confidence of parents in parenting and coping. Sessions cover advice on parenting, bonding and mother–infant interaction and relationship, partner and family support, and brief psychological coping strategies. The subsequent four sessions are designed to be delivered over the telephone, which is suitable for an inpatient setting when participants may be discharged or on home leave from the unit in preparation for discharge; they can also be delivered face-to-face. These sessions allow the participant to review progress and practice skills, thereby enhancing her parenting confidence, competence and overall wellbeing.

The intervention has a flexible delivery format, which is suitable for mothers with severe mental health. Sessions can be delivered flexibly to allow participants to attend to their baby’s needs, if required, or to pace the sessions in line with their own mental health needs. Session length can range from 45 to 90 min for the initial four sessions and 30–60 min for the telephone sessions. The content of one Baby Triple P session can also be covered in two briefer sessions and breaks can be taken.

### Intervention fidelity

Two therapists (an occupational therapist and a clinical psychologist), one from each MBU, have been trained by an accredited and experienced Baby Triple P trainer to deliver the intervention. Both therapists will work to the established Baby Triple P protocol and manual and record any deviations. Existing Baby Triple P fidelity measures [44] will be completed to assess treatment fidelity. Some sessions (approximately 5–10%) will be digitally recorded (where feasible) with participant consent and subjected to independent therapy fidelity checking by listening to the recordings to ensure the Baby Triple P protocol is being adhered to. During the intervention phase, both therapists will use the Triple P model of peer supervision [52].

### Treatment as usual (TAU)

As it is anticipated that TAU will vary based on patient need, ward acuity and staff preferences [21, 53], TAU offered to MBU patients over the study duration will be recorded. In line with all standard and individually prescribed clinical interventions as directed by the MBU clinical teams, TAU usually consists of case management using a care programme approach from their allocated MBU psychiatric nurse, pharmacological interventions from psychiatry and non-parenting, psychological interventions (e.g. cognitive behavioural therapy for depression). Nursery nurses and MBU staff also offer psychosocial interventions (e.g. Baby Massage or Video Interactive Guidance) to inpatients, which participants can choose to engage in, but these are not structured, manualised activities that focus on improving parenting competence [18, 21]. TAU will vary depending on patient needs, capacity and preferences [21]. Following MBU discharge, TAU will typically include multidisciplinary team management offered by (perinatal) community mental health teams or crisis or home treatment teams.

Participants allocated to TAU will be offered the Baby Triple P Workbook upon completion of the study (i.e. at the 6-month follow-up assessment).

### Outcomes and measures

Outcomes relate to uptake (the proportion of eligible participants consenting to partake), attrition and retention rates. Through semi-structured interviews with participants and MBU staff we will also further explore the acceptability of the study procedures and intervention. We will assess the feasibility and acceptability outcomes as follows:

1. Feasibility of recruitment will be measured through recording how participants found out about the study, recruitment rates, number of potential participants found eligible, initially approached by MBU staff, consented to be approached by the research team, and approached about the study by the research team, reasons for declining to be approached or take part in the study if known, and percentage of eligible participants consented and their characteristics.
2. Feasibility of randomisation will be assessed by recording number of refusals to be randomised.
3. Engagement/retention in the study and to study procedures will be measured by recording the number of participants who withdraw and the time point at which they withdraw from the study. Reasons for leaving the study early will be recorded if known. The number of participants who complete outcome measures at each of the time points and the completeness of their data will be recorded.
4. Engagement/retention in the intervention will be measured by recording the percentage of intervention sessions delivered, duration and maternal self-reports of time spent between sessions on Baby Triple P as well as maternal use of the workbooks. Reasons for leaving the intervention early will be recorded if known.
5. Acceptability of the intervention and study design and procedures will be assessed post-intervention via a questionnaire (for acceptability of the intervention only) and semi-structured interviews with participants allocated to receive Baby Triple P plus TAU (*n* < 15) and MBU staff (*n* ≥ 15).

Self-report and observer-rated measures will be used alongside routinely collected information such as psychiatric history. Participants will be asked to complete an adapted version of the Family Background Questionnaire [54] asking about family sociodemographic information, family composition and maternal social support, past psychiatric and admission history, symptom presentation, diagnosis and pregnancy experiences. Data on medication type and use will be collected alongside baby care information from nursery nurses’ and clinical case-notes. Social services involvement and safeguarding information will also be collected. We will record this if it is the participant’s first admission.

Baseline assessments will be conducted prior to the start of the Baby Triple P plus TAU or TAU phase by a researcher face-to-face with participants at the MBU (referred to as Time 1). Post-intervention assessments will be conducted after the intervention phase (which will be 10 weeks (plus or minus a week) post-baseline assessments; Time 2) and at a final follow-up assessment at 6 months (26 weeks plus or minus a week, post-baseline assessments; Time 3). They will be conducted by a researcher in person at a location convenient for participants (e.g. at home or intervention setting) or at the MBU (if the participant has not been discharged) and/or over the telephone and/or via post. A flexible assessment method was chosen deliberately in order to be responsive to the needs of this population and their preferences.

Participants will be asked to complete the following self-report measures at all assessment time-points (all measures will be presented to participants in a booklet (for ease of completion) and hence the order of the questionnaire presentation will be the same for all assessments and for both MBUs):

1. The Maternal Efficacy Questionnaire (MEQ) [55] to assess maternal confidence and competence as an indicator of overall wellbeing. Participants are asked to rate 10 questions on a 4-point Likert scale and to consider how they rate themselves in comparison to other mothers. The MEQ is a domain-specific measure of parental self-efficacy because nine of the 10 items address a mother’s self-efficacy in specific parenting tasks (e.g. soothing the baby; feeding, changing, and bathing the baby) and one item assesses general parental self-efficacy. The scale has good internal reliability (Cronbach’s alpha = 0.86) and is strongly correlated with the PSI Sense of Competence Scale (*r* = − 0.75, *p* < 0.001).
2. The Brief Depression, Anxiety and Stress Scale (DASS) [56, 57] to assess mood. Participants rate 21 statements on a 4-point Likert Scale (ranging from 0 to 3). Internal consistencies (coefficient alpha) for each scale for the DASS normative sample were determined as Depression 0.91, Anxiety 0.84, Stress 0.90 [56]. The DASS-21 has good internal reliability with Cronbach’s alphas of 0.88 (95% confidence interval (CI) ¼ 0.87–0.89) for the Depression subscale, 0.82 (95% CI ¼ 0.80–0.83) for the Anxiety subscale, 0.90 (95% CI ¼ 0.89–0.91) for the Stress subscale, and 0.93 (95% CI ¼ 0.93–0.94) for the Total scale [57].
3. The Postpartum Bonding Questionnaire (PBQ) [58] to assess the mother–infant relationship. Participants rate 25 statements on a 6-point Likert scale, which can be broken down into four subscales (indicating (1) general emotional bond and response to the baby, (2) feelings of anger and rejection, (3) confidence in caring for the baby and (4) possible risk to the baby). Low scores indicate good overall bonding. Its test-retest reliabilities (Pearson’s r) were of 0.95, 0.95, 0.93 and 0.77, respectively, in a sample of 30 mothers. PBQ has acceptable internal reliabilities with Cronbach’s alphas of 0.79 for impaired bonding, 0.63 for rejection and anger, and 0.63 for anxiety about care. Due to zero variance in the two items of the risk of abuse scale, the internal consistency of risk of abuse could not be determined. When these items were omitted from the total PBQ scale, Cronbach’s alpha was 0.77 [59].
4. The Brief Symptom Inventory [60] to assess symptom severity and distress. This 53-item inventory asks participants to rate the extent to which they have been bothered (0 = not at all to 4 = extremely) in the past week by various symptoms. The Brief Symptom Inventory has nine subscales designed to assess individual symptom groups and three global indices (such as the Global Severity Index, the Positive Symptom Distress Index and the Positive Symptom Total Scale). Good internal consistencies (Cronbach’s alphas) for subscales were reported as between 0.71 (psychoticism) and 0.85 (depression) in a sample of 719 psychiatric patients. Good test-retest reliability scores (Pearson r) were reported for the Global Severity Index (*r* = 0.90), the Positive Symptom Distress Index (*r* = 0.87) and the Positive Symptoms Total Scale (*r* = 0.80) [61].
5. The EQ-5D-5 L [62] to measure health status (in the domains of mobility, self-care, usual activity, pain/distress, and anxiety and depression). Quality-adjusted life years will be estimated from the EQ-5D as recommended by NICE for the economic evaluation of healthcare interventions [63].
6. A bespoke health and social care resource use questionnaire to capture services used during the study, particularly following MBU discharge. It takes 5–10 min to complete. At 10 weeks post-baseline, participants will be asked to report on health and social services they have accessed since entering the study. At 6 months post-baseline, they will be asked to report on the health and social services they have accessed since the 10 weeks post-baseline assessment.

The following measures will be completed by MBU staff, if feasible, at assessment time-points or weekly if this is MBU routine practice:

1. The Health of the Nation Outcome Scales [64] will be used to assess health outcomes. Clinical staff rate service users’ difficulties in 12 areas using a 4-point Likert scale (from ‘not a problem’ to being ‘a severe problem’). They are designed for repeated use to capture changes in a wide range of health and social domains, including psychiatric symptoms, physical health, functioning, relationships and housing. The areas are (1) overactive, aggressive, disruptive or agitated behaviour, (2) non-accidental self-injury, (3) problem drinking or drug-taking, (4) cognitive problems, (5) physical illness or disability problems, (6) problems associated with hallucinations and delusions, (7) problems with depressed mood, (8) other mental, behavioural social problems, (9) problems with relationships, (10) problems with activities of daily living, (11) problems with living conditions and (12) problems with occupation and activity.
2. The Clinical Global Improvement Scale (CGI) [65] will be used to gauge improvements from admission to discharge. The CGI is a commonly used measure of symptom severity, treatment response and the efficacy of treatments in treatment studies of patients with mental health difficulties. The severity scale (CGI-S) is a 7-point scale that requires the clinician to rate the severity of the patient’s illness at the time of assessment, relative to the clinician’s past experience with patients who have the same diagnosis. Scores range from ‘not at all ill’ to ‘extremely ill’. The Improvement scale (CGI-I) is a 7-point scale that requires the rater to assess how much the patient’s illness has improved or worsened relative to a baseline state at the beginning of the intervention. Ratings range from ‘very much improved’ to ‘very much worse’. The CGI Efficacy Index is a 4-point × 4-point rating scale that assesses the therapeutic effect of the treatment ranging from ‘unchanged to worse’ to ‘do not significantly interfere with patient’s functioning’.
3. The Brief Psychiatric Rating Scale [66] is a scale which a clinician or researcher may use to measure 24 symptoms, including depression, anxiety, hallucinations and unusual behaviours. Each symptom is rated on a 7-point Likert scale (from not present to extremely severe); it is commonly used in psychiatric settings.
4. The Louis MACRO (Mother and Child Risk Observation) Measure [67] will be used to assess infant wellbeing and the mother–infant relationship. Using a 5-point Likert scale, MBU staff rate mothers on 10 items across five domains (including safety, care, emotional responsiveness, mother’s mental stage and infant characteristics that may contribute to parenting difficulties). The Louis MACRO has excellent internal reliability with Cronbach’s alphas of 0.96 for the total scale and between 0.79 and 0.95 for the 10-item subscales. Its subscales’ inter-rater reliabilities (Pearson’s r) were between 0.81 and 0.93 and its test-retest reliability scores were 0.71 to 0.93. Note that this measure is routinely used by some MBUs.

### Assessment of acceptability of intervention and study procedures

Acceptability will be examined quantitatively through study uptake, attrition and retention rates as well qualitatively through interviews because participants’ experiences of participation in a trial and its procedures (e.g. quantity and frequency of assessments, randomisation process) are important for informing the design and methods of a future full-scale RCT. In addition, the acceptability of the intervention will be assessed through a validated satisfaction questionnaire [68] and through qualitative research interviews (described below) in order to examine if the current intervention is considered to be acceptable and appropriate for this population.

The Client Satisfaction Questionnaire [68] will be used to collect information on participants’ views on the acceptability of Baby Triple P and their overall satisfaction with this intervention. Participants are asked to rate 13 questions (e.g. to what extent has the programme met your needs?) on a 7-point Likert scale and provide answers to three open-ended questions (e.g. do you have any other comments about this programme?). This will be administered to participants (allocated to receive Baby Triple P plus TAU) in person or over the telephone or via post after they have completed the intervention.

Semi-structured individual interviews will be conducted post-intervention with MBU staff and trial participants allocated to the intervention arm prior to or around the time of their final follow-up assessment. All participants will be given a separate participant information sheet and consent form. Staff participants will be recruited through an advert or poster displayed on the ward. All interviewees will be interviewed at a time and place convenient to them or via telephone for up to 90 min. Interviewees will be given the choice to be interviewed by a researcher who works on the study at the same MBU they were admitted to and who they know or by a researcher who works at a different MBU and who they do not know. This is to ensure that participants feel comfortable expressing their views in the interview.

Interviews with participants and MBU staff will explore (1) acceptability, usefulness and user-friendliness of the intervention, (2) changes in and any benefits to themselves since receiving the intervention, (3) perceived changes/improvements to the intervention needed, (4) ways to increase adherence to and engagement in the intervention, (5) views on the procedures of the trial, and (6) perceived changes/improvements needed to increase adherence to and engagement in the study.

### Data analysis

Participant flow will be reported in accordance with the CONSORT statement (Fig. 1). Baseline data will be described using summary statistics (means and standard deviations or numbers and percentages). The feasibility findings will be tabulated and associated graphical summaries of the key indicators of success will be presented. All anonymised participant data will be included in the efficacy analysis using an intention-to-treat approach.

Data from clinical outcome measures will be analysed using a linear regression model, allowing for baseline measurements of outcome, treatment assignment and NHS trust as covariates, at each assessment point separately. We will report 95% CIs and point estimates for effects instead of *p* values. The results will be used to provide an indication of the population effect size required for a future full-scale definitive trial. However, as a feasibility RCT, this study is not powered to find an effect.

### Economic evaluation

To determine the feasibility of conducting a full economic evaluation, data required to identify key drivers of costs associated with the Triple P intervention and TAU will be collected. This will include the resources required to deliver the intervention and NHS and social care services (inpatient, outpatient, primary and community care services) used by participants in both treatment groups during the study follow-up period. The EQ-5D-5 L [62] will be used to estimate quality-adjusted life years based on health status and associated utility tariffs, as per the approach recommended by NICE at the time of the analysis [6]. The quality and completeness of resource use and health status data will be reported. Utility values will be compared with scores on the MEQ [55] to determine whether the characteristics (e.g. direction) of any observed health outcomes are comparable according to both measures.

### Qualitative data analysis

All interviews will be digitally recorded, transcribed verbatim and subject to framework analysis to allow both inductive and deductive coding [69]. An initial coding framework will be developed to reflect key service and service user-determined topics covered by the interview schedule. Following data familiarisation, this framework will be augmented and extended to encompass new emerging themes. Coding will be undertaken by the first author and/or a member of the research team, experienced and trained in qualitative data analysis, and in consultation with our service user consultants to capture potentially different perspectives.

Initially, a subset of transcripts will be coded independently and findings will be discussed to develop a shared theoretical framework between academic and service user researchers. This framework will then be applied to the remaining transcripts. As the constant comparison of new data occurs, the framework will be amended and refined to enable the introduction of new codes or the deletion of redundant codes. Data will be interpreted and analysed within the final framework to structure and compare participant and staff views about the intervention.

Analysis will be overseen by research team members with qualitative data analysis experience. Regular meetings will ensure that the emerging codes are grounded in the original data. The trustworthiness of the final analysis will be enhanced through the integration of data from different stakeholders and through researcher triangulation.

## Discussion

This will be the first study to examine the feasibility and acceptability of conducting a RCT of a manualised parenting and psychological intervention for women admitted to one of two inpatient MBUs. It is also the first feasibility RCT to examine the possible benefits of a manualised parenting intervention in an acute inpatient setting with this population [18, 70]. The intervention is first offered in the MBU and, depending on admission time, it continues following discharge, offering continuity of care to participants whose care has to be transferred from inpatient acute psychiatric care to community care following MBU discharge. It is anticipated that routine post-discharge care will vary depending on local psychiatric and psychological arrangements and support.

This study allows for the exploration of pragmatic issues that will inform a larger RCT. This feasibility study will allow us to assess the suitability of the Baby Triple P programme for this clinical population and, if necessary, identify how it can be refined and tailored to meet participant capabilities and needs during an inpatient admission in terms of both its content and the practicalities of its delivery (e.g. by refining the length and number of sessions per week and/or mode of delivery). As participants will present with a range of perinatal mental health diagnoses and difficulties as well as parenting concerns and levels of confidence, the appropriateness of the content and practicalities of attending sessions will be examined in interviews.

The information gained from this study will provide an indication of what recruitment methods work or do not work in this setting and with this clinical population, enabling us to identify any participant-, staff- or service-related barriers to recruitment alongside solutions to overcoming these. It will help us to determine whether it is possible to recruit a large enough sample for a full-scale RCT and in what timescale. In addition, the adequacy of the recruitment sites can be examined in order to determine the characteristics of the sites that make them most suitable for a future RCT of this intervention. The inclusion and exclusion criteria will be scrutinised to determine whether it affords a large enough and suitable sample and whether revisions are needed for a future RCT (e.g. to eligibility criteria, etc.). An exploration of participant and staff facilitators for and barriers to study engagement is likely to highlight practicalities that need to be considered when embedding a RCT in this setting.

Inpatient psychiatric wards can be challenging environments for patients to recover in and for staff to work in. Ward acuity and severity and expression of mental health problems alongside staffing issues can influence the availability of routine psychosocial and psychological support for women admitted to a MBU, whilst the baby’s needs also determine if the mother can access any support being offered. Hence, it is anticipated that TAU across both MBUs and outpatient routine care will vary. As evidence of cost-effectiveness is essential to inform future NHS policy, this study will determine the best approach to obtain the data needed to conduct a robust economic evaluation of the intervention in a full RCT. Furthermore, this study will allow us to collect data on what aspects of ward activities have to be considered in order to identify the best approaches (e.g. the recruitment procedures, the completion of measures, the intervention delivery and/or format) to implement this intervention successfully.

It will be vital to identify the most appropriate, effective and sensitive outcome measures in order to detect meaningful participant reported and observer rated changes in parent and infant wellbeing resulting from the intervention. The use of observer rated measures is indicated given that participants are admitted to an inpatient unit due to a crisis that may affect their ability to concentrate on and complete self-report measures, but training is required to achieve inter-rater reliability across different raters. This study will enable us to establish which assessments are meaningful, relevant and appropriate during inpatient admission and after discharge.

The inclusion of service user consultants in various aspects of this study has been emphasised. As part of this, we will also assess the type of training and amount of support required for service user participation in the analysis of interview data and other study aspects. Staff views on the use of the intervention and the impact of the study on the MBU in combination with participant experiences will inform the development of a future RCT.

### Dissemination policy

The research team intend to disseminate outcomes from this study in peer-reviewed open access journals and at relevant conferences. Participants will be provided with a lay summary of the findings if they indicated that they wish to receive this information.

### Trial status

Recruitment started in November 2016 and is ongoing.

## Additional file

Additional file 1: SPIRIT 2013 Checklist: Recommended items to address in a clinical trial protocol and related documents. (DOC 122 kb)

## Abbreviations

CGI: The Clinical Global Improvement Scale
CTU: Clinical Trials Unit
DASS: Brief Depression, Anxiety and Stress Scale
EQ-5D-5L: Five level EQ-5D
MBU: Mother and Baby Unit
MEQ: Maternal Efficacy Questionnaire
NHS: National Health Service
NICE: The National Institute for Health and Care Excellence
PBQ: Postpartum Bonding Questionnaire
RCT: randomised controlled trial
SMI: severe mental illness
TAU: treatment as usual
