# Correction to: Enhancing maternal and infant wellbeing: study protocol for a feasibility trial of the Baby Triple P Positive Parenting programme for mothers with severe mental health difficulties (the IMAGINE study)

**DOI:** 10.1186/s13063-018-2918-7

**Published:** 2018-09-21

**Authors:** Anja Wittkowski, Kim Cartwright, Richard Emsley, Penny Bee, Elizabeth Camacho, Rachel Calam, Catherine Cross, Kathryn M. Abel, Holly Reid

**Affiliations:** 1 Division of Psychology and Mental Health, School of Health Sciences, Faculty of Biology, Medicine and Health, The University of Manchester, Manchester Academic Health Science Centre, Zochonis Building, Brunswick Street, Manchester, M13 9PL UK; 2 Department of Clinical Psychology, Greater Manchester Mental Health NHS Foundation Trust, Laureate House, Wythenshawe Hospital, Southmoor Road, Manchester, M23 9LK UK; 3 Department of Biostatistics and Health Informatics, King’s College London, Institute of Psychiatry, Psychology & Neuroscience, De Crespigny Park, London, SE5 8AF UK; 4 Division of Nursing, Midwifery & Social Work, The University of Manchester, Jean MacFarlane Building, Oxford Road, Manchester, M13 9PL UK; 5 Manchester Centre for Health Economics, Division of Population Health, Health Services Research, and Primary Care, The University of Manchester, Jean Macfarlane Building, Oxford Road, Manchester, M13 9PL UK

## Correction

Following publication of the original article [1], the authors reported that Elizabeth Camacho was omitted from the author name list. In this Correction the author and her affiliation are reflected.

The original publication of this article has been corrected.
